# Extracellular Metabolites from *Saccharomyces cerevisiae* Modulate the Growth and Fermentative Performance of *Kluyveromyces marxianus*

**DOI:** 10.3390/microorganisms14040890

**Published:** 2026-04-16

**Authors:** Jairo Gallardo-Rivera, Oscar E. Soto-Malpica, Erick D. Acosta-García, Perla G. Vázquez-Ortega, Juan A. Rojas-Contreras, Nicolas O. Soto-Cruz

**Affiliations:** Departamento de Ingenierías Química y Bioquímica, Tecnológico Nacional de México, Instituto Tecnológico de Durango, Blvd. Felipe Pescador 1830 Ote., Durango 34080, Mexico; 23041391@itdurango.edu.mx (J.G.-R.); 20040755@itdurango.edu.mx (O.E.S.-M.); 20040540@itdurango.edu.mx (E.D.A.-G.); pvazquez@itdurango.edu.mx (P.G.V.-O.); jrojas@itdurango.edu.mx (J.A.R.-C.)

**Keywords:** yeast–yeast interaction, cell-free supernatant, growth inhibition, *S. cerevisiae* autolysis, volatilome

## Abstract

During alcoholic fermentations, some non-*Saccharomyces* yeasts are often displaced by *Saccharomyces cerevisiae*. It remains unclear whether this displacement is mediated by metabolites produced by *S. cerevisiae* or depends on cell–cell contact. This study evaluated the effects of extracellular metabolites produced by *S. cerevisiae* on the growth and fermentative performance of *Kluyveromyces marxianus* isolated from mezcal fermentations. The development of both yeasts was evaluated in monocultures and in co-cultures with physical contact. Indirect interaction was also tested by exchanging cell-free fermented media. The growth and fermentative response of *K. marxianus* in cell-free *S. cerevisiae*-fermented medium showed modulation that depended on the growth phase during which the exchange was performed. The exchange performed at 6 h (exponential phase) limited the maximum growth of *K. marxianus* and resulted in lower fermentative performance. When the exchange was done during the stationary phase (17.5 h), *K. marxianus* exhibited a longer stationary phase and better fermentative performance. Finally, when the exchange was performed at 24 h (the beginning of the death phase), the effects on survival and fermentative performance were less pronounced. Furthermore, co-culture with cell–cell contact showed that direct competition and/or mechanisms dependent on physical contact intensify the displacement of *K. marxianus*. The results show that direct cell–cell contact promotes greater inhibition of *K. marxianus* by *S. cerevisiae*, which is relevant for the design of mixed fermentations aimed at achieving a greater contribution of non-Saccharomyces yeasts to the organoleptic characteristics of alcoholic beverages.

## 1. Introduction

Spontaneous fermentation to produce traditional beverages such as mezcal and tequila is a complex microbial process involving indigenous yeasts, both *Saccharomyces* and non-*Saccharomyces* [1,2,3]. Yeast diversity is crucial for the formation of compounds that determine the sensory characteristics of the final product, both in agave-based beverages [1,4] and in wine production [5].

Consequently, the use of mixed starter cultures including *Saccharomyces cerevisiae* and non-*Saccharomyces* strains has been proposed [2]. However, in mixed fermentations, microbial succession occurs as different species adapt to changing environmental conditions, resulting in a dynamic growth pattern [6]. The loss of cultivability of non-*Saccharomyces* species is considered a multifactorial phenomenon, associated with physicochemical changes in the medium, such as increased ethanol, decreased pH, and nutrient depletion [7], and with possible interspecific interactions among yeasts [8,9]. In recent years, interest in understanding the decline in non-*Saccharomyces* species has grown, especially following discoveries linking this loss to the action of extracellular compounds released by *S. cerevisiae* during its interactions with other yeasts [10]. Various substances, such as ethanol, short-chain fatty acids, killer proteins, antimicrobial peptides (AMPs), and sulfur dioxide (SO_2_), are produced by *S. cerevisiae* and can act as selective growth-limiting factors against non-*Saccharomyces* species [5,9,11].

Both direct and indirect interactions negatively impact non-*Saccharomyces* yeasts by interfering with their development and metabolic performance [12]. Therefore, it is important to understand the interactions between *S. cerevisiae* and non-*Saccharomyces* species to identify the possible mechanisms underlying this inhibition. Some non-*Saccharomyces* yeasts, such as *Kluyveromyces marxianus*, are notable for their production of esters, terpenes, and higher alcohols. *Kluyveromyces marxianus* is considered the predominant yeast population in mezcal fermentation processes [13,14] and is valued for its ability to produce a wide range of bioactive compounds with industrial and biotechnological applications [14].

The objective of this research was to determine the inhibitory effect of extracellular compounds produced by *S. cerevisiae* on the growth and fermentative yield of *K. marxianus*. Specifically, it was evaluated whether these compounds exhibit an inherent antagonistic effect or whether their production depends on a prior chemical interaction between the two species.

## 2. Materials and Methods

### 2.1. Microorganisms and Inoculum Preparation

The yeast strains were previously isolated from *Agave durangensis* musts produced by artisanal mezcal factories in the state of Durango, Mexico. *Saccharomyces cerevisiae* ITD-00185 was isolated from El Mezquital municipality [13] while *Kluyveromyces marxianus* ITD-01005 is a strain from Durango municipality [15]. They are kept preserved in 30% (*v*/*v*) glycerol at −20 °C at the strain collection of the Microbial Biotechnology Laboratory of the TecNM/Technological Institute of Durango.

Once reactivated by flash thawing in a 25 °C water bath, the yeast cells were plated and incubated at 28 °C onto YPD agar plates. The solid YPD medium contained, per liter: yeast extract, 10 g; peptone, 20 g; dextrose, 20 g; and agar, 20 g. Precultures were prepared by transferring an isolated colony from an agar plate to 50 mL of liquid YPD medium. Incubations were carried out for 12–14 h at 28 °C with shaking at 150 rpm in 150 mL Erlenmeyer flasks sealed with a cotton plug. Subsequently, the cells were washed with 100 mM phosphate-buffered saline (PBS) (pH 4.5). The cell density was adjusted to prepare the inoculum to 1 × 10^6^ cells/mL for monocultures and to 0.5 × 10^6^ cells/mL for co-cultures, with each species.

### 2.2. Monocultures and Co-Cultures

Monocultures and co-cultures were carried out in 250 mL Erlenmeyer flasks containing 150 mL of medium. The semi-synthetic medium M2 supplemented with casein peptone was used. It was formulated to simulate the concentration and proportions of hexoses (fructose and glucose) present in diluted agave must employed in artisanal fermentation processes [16]. This medium contained, per liter: glucose, 10 g; fructose, 90 g; yeast extract, 1 g; (NH_4_)_2_SO_4_, 2 g; MgSO_4_·7H_2_O, 0.4 g; KH_2_PO_4_, 5 g; and casein peptone, 2 g/L. The sugars were sterilized separately from the other components, in both cases at 121 °C for 15 min. The initial pH was adjusted to 5.0.

Fermentations were performed in triplicate for all conditions, and samples (1 mL) were taken every 3 h for the first 30 h, and subsequently every 6 h until 48 h of incubation were completed. The samples were centrifuged at 6720× *g* for 3 min, filtered through nylon membrane discs (0.45 μm), and stored at −20 °C until analysis [15].

Monocultures of *S. cerevisiae* and *K. marxianus* were carried out in 250 mL Erlenmeyer flasks containing 150 mL of M2 medium. Each strain was inoculated separately at an initial density of 1 × 10^6^ cells/mL. The cultures were incubated at 28 °C with constant shaking at 150 rpm for 48 h and used as reference controls for each species. To evaluate the effect of direct physical interaction between the yeasts, co-cultures of *S. cerevisiae* and *K. marxianus* were performed under the same medium and culture conditions. In this case, the initial cell density was adjusted to 0.5 × 10^6^ cells/mL for each species. The co-cultures were incubated at 28 °C and 150 rpm for 48 h.

### 2.3. Cultures for Indirect Interaction Assays

**Figure 1:**
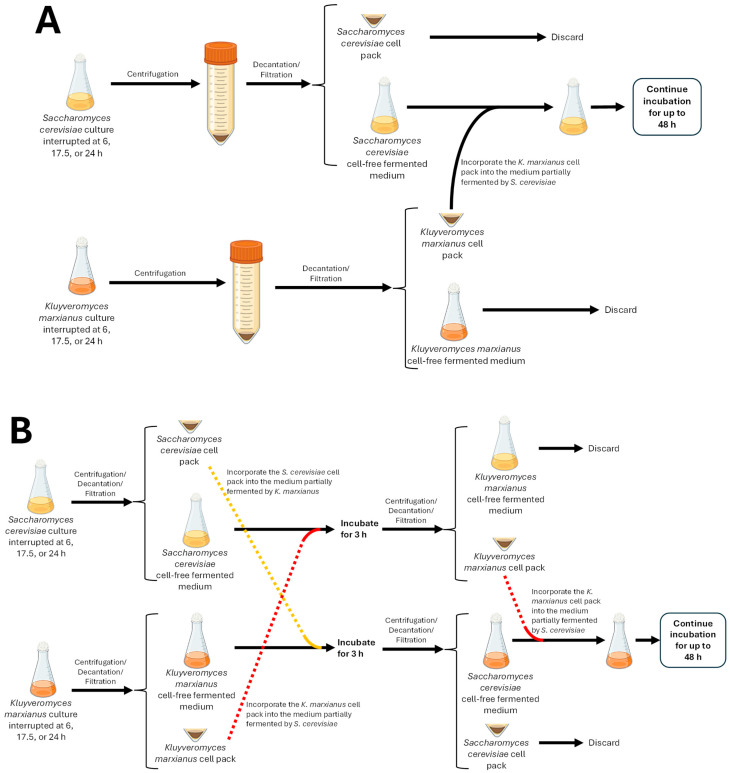
Experimental strategy to perform the cultures of *Kluyveromyces marxianus* in a cell-free medium partially fermented by *Saccharomyces cerevisiae* without (**A**) and with (**B**) cross exposure to metabolites produced by the opposite yeast.

These fermentations allowed us to evaluate the effects of extracellular compounds produced by *S. cerevisiae* on the growth and fermentative capacity of *K. marxianus* at the exponential phase, the stationary phase, and the onset of the death phase. It was done in the absence of direct physical contact, using exchange between cell-free fermented media, as illustrated in Figure 1.

Parallel and independent fermentations of *S. cerevisiae* and *K. marxianus* were performed in 250 mL Erlenmeyer flasks containing 150 mL of M2 medium, incubated at 28 °C with constant shaking at 150 rpm. Considering the growth kinetics in monoculture, three fermentation interruption times were selected. These times corresponded to specific phases of the *S. cerevisiae* growth curve: exponential phase (6 h), stationary phase (17.5 h), and the beginning of the death phase (24 h). In all cases, fermentations were performed in triplicate, with samples collected every 3 h during the first 30 h of incubation and every 6 h thereafter until a total of 48 h of incubation was completed. As the procedure was similar in all three cases, only the exchange procedure during the exponential phase is described below.

Separately, under aseptic conditions, the parallel cultures were centrifuged at 3354× *g* for 7 min at 4 °C after 6 h of incubation (Figure 1A). The supernatant from the *K. marxianus* culture was discarded, and the cell pack was washed twice with sterile phosphate-buffered saline (PBS). On the other hand, after decantation, the cell pack of *S. cerevisiae* was discarded. The supernatant was filtered through a 0.45 µm nylon membrane to obtain a cell-free supernatant, the sterility of which was verified by plating onto YPD agar plates. This supernatant was used directly, without modification, to resuspend the cell pack of *K. marxianus* and allow this yeast to continue consuming the sugars that *S. cerevisiae* left unfermented. The incubation continued at 28 °C and 150 rpm for a total of 48 h. In this way, the *K. marxianus* cells were exposed to the extracellular compounds produced by *S. cerevisiae*, without cell–cell contact. To ensure the exclusive presence of *K. marxianus* after the medium exchange, spread cultures were performed on the differential medium Wallerstein Laboratory Nutrient Agar (WLN, Sigma-Aldrich, St. Louis, MO, USA) from samples taken immediately after the exchange and at the end of the incubation period (48 h).

As shown in Figure 1B, in the cross-exposure assays for metabolites produced by the opposing strain, after 6 h of incubation, the parallel cultures were centrifuged at 3354× *g* for 7 min at 4 °C. The supernatants were then separated from the cell packs. The supernatant fermented by *S. cerevisiae* was used to resuspend the *K. marxianus* biomass, and the *S. cerevisiae* cell pack was resuspended in the supernatant fermented by *K. marxianus*. The flasks were incubated for 3 h at 28 °C and 150 rpm, and the separation process was repeated, returning each species to its original medium. Subsequently, the fermentations were resumed under the same temperature and agitation conditions until a total of 48 h of incubation was completed. The sterility of the cell-free supernatants was verified by plating on YPD agar plates. Once the inoculations were carried out, the cultures’ purity was verified by plating in WLN medium to confirm the exclusive presence of each species.

### 2.4. Analytical Techniques

At each sampling time, cell density was determined by direct counting in a Neubauer chamber, using appropriate dilutions of the sample in sterile isotonic saline (0.9% *w*/*v*). Serial dilutions were then performed to obtain an appropriate concentration that yielded between 30 and 300 colonies per plate for viable count determination. The diluted suspensions were spread onto Petri dishes containing WLN medium. Plates were incubated at 28 °C for 24–36 h, until well-defined colonies appeared. Results are reported in colony-forming units (CFU/mL) expressed in logarithmic cycles.

For the quantification of glucose, fructose, and ethanol, an Agilent Technologies 1200 HPLC system (Agilent, Santa Clara, CA, USA) equipped with a refractive index detector was used. Calibration curves were constructed for each analyte using external standards. One microliter of sample was injected for compound separation into an Aminex^®^ (Dublin, Ireland) HPX-87H+ column (300 mm × 7.8 mm) under the following conditions: column temperature 65 °C, refractive index detector temperature 60 °C, and mobile phase 0.5 mM sulfuric acid (Sigma-Aldrich, Burlington, MA, USA) at a constant flow rate of 0.6 mL/min [15].

The protein profile of cell-free supernatants from *S. cerevisiae* monocultures in M2 medium was analyzed at 6, 17.5, and 24 h of fermentation. Unfermented M2 medium was also analyzed as a control. Proteins were precipitated using the methanol-chloroform method [17]. Briefly, 3 mL of the sample was added to 12 mL of methanol and vortexed. Then 3 mL of chloroform was added, and the mixture was vortexed again. Afterward, 9 mL of distilled water was added, and the mixture was homogenized. The mixture was centrifuged for 15 min at 3350× *g*, the upper aqueous phase was removed, and the protein material located at the interface was recovered. Twelve milliliters of methanol were added, the mixture was vortexed, and the mixture was centrifuged for 30 min at 3350× *g*. The supernatant was removed without disturbing the protein pellet, which was allowed to dry completely in a fume hood and resuspended in 20 µL of phosphate-stabilized saline (PBS).

Electrophoretic separation was performed in a polyacrylamide gel (SDS-PAGE) under denaturing conditions [18], using a Laemmli-type batch system in a vertical chamber (BIO-RAD Mini PROTEAN^®^ Tetra System, Hercules, CA, USA). 15% and 20% gels (polyacrylamide-bisacrylamide) were used. Fifteen milliliters of each sample, precipitated and resuspended in PBS, were loaded onto the gel. For molecular weight estimation, samples were run in parallel with a molecular weight marker (PageRuler^TM^ Unstained Protein Ladder, 10–200 kDa). After running, the gels were fixed for 2 h in a mixture of methanol:glacial acetic acid:formaldehyde (6:4:0.5, *v*/*v*/*v*) and stained with AgNO_3_.

The volatile compounds present in cell-free extracts of *S. cerevisiae* were extracted by headspace solid-phase microextraction (HS-SPME) and analyzed by gas chromatography-mass spectrometry (GC-MS), following the procedure described previously [19]. Briefly, 5 mL of the sample was placed in a microextraction vial containing 1.5 g of NaCl. 50 µL of a 1-pentanol solution (300 mg/L) was added as an internal standard for the semi-quantification of volatile compounds. The mixture was incubated for 5 min at 35 °C. Subsequently, a 50/30 µm DVB/CAR/PDMS SPME fiber (Supelco, St. Louis, MO, USA) was exposed to headspace extraction for 60 min at 35 °C. The fiber was then inserted into the injection port of an Agilent 7890A gas chromatograph, where the compounds were thermally desorbed for 15 min at 250 °C.

Chromatographic separation was performed on an FFAP column (30 m × 0.32 mm × 0.25 µm). Ultra-high-purity helium was used as the carrier gas at 1 mL/min. The oven temperature was set to 40 °C for 3 min, followed by a ramp of 3 °C/min to 52 °C, which was held for 1 min. A ramp of 10 °C/min was then used to increase the temperature to 200 °C, which was held for 15 min. The mass spectrometer (Agilent 5975C) was operated at 230 °C at 70 eV in SCAN mode (1.6 scans/s). Mass spectra were deconvolved using AMDIS software (v. 2.73, build 149.31) and compared with the NIST 2011 library, accepting ≥80% matches. Additionally, linear retention indices were calculated according to the Van den Dool & Kratz [20] method using a homologous series of n-alkanes (C7–C30). These values were compared with those reported in the NIST Chemistry WebBook database (https://webbook.nist.gov/chemistry/name-ser/, accessed on 24 March 2026).

The semi-quantification of each compound was estimated as 1-pentanol equivalents, based on the ratio of the analyte area to the internal standard area. Chromatograms were processed using OpenChrom^®^ software v. 1.6.8, applying baseline correction, peak detection using the first derivative algorithm with valley detector (threshold: low; window size: 5; filter mode: exclusive), and area integration by the trapezoidal method (scale factor: 1.0; area constraint enabled). The assignment of compounds was supported by the calculation of retention indices and an identification template based on retention time windows.

### 2.5. Fermentation Parameters Calculation

The Gompertz equation (Equation (1)) was fitted to the growth data collected between 0 and 18 h of incubation using the Solver tool in Excel (Microsoft Corporation, Redmond, WA, USA). Subsequently, the fitted parameters were used to calculate the specific maximum growth rate and the lag time as described previously [21].

(1) $$X\left(t\right)=a\cdot e^{-e^{\left(b-c\cdot t\right)}}$$

(2) $$\mu_{max}=\frac{c\cdot a}{e}$$

(3) $$t_{lag}=\frac{b-1}{c}$$

where X(t) is the cell density (LOG CFU/mL);

a (LOG CFU/mL), b (dimensionless), and c (h^−1^) are adjustment parameters of the Gompertz equation;

t is the incubation time (h);

$\mu_{max}$ is the specific maximum growth rate (h^−1^), which is the derivative at the inflexion point of the Gompertz equation;

$t_{lag}$ is the lag time (h), i.e., elapsed time between inoculation and the start of exponential growth, which is the intercept on the abscissa axis of the tangent that passes through the inflexion point.

The yield of ethanol produced with respect to the sugars consumed (*Y_P/S_*, g/g) was calculated as the average of the ratios (g ethanol produced/g sugar consumed) corresponding to the last three incubation times.

### 2.6. Statistical Analysis

A one-way analysis of variance (*p* < 0.05) with Tukey’s test was used to compare the means between the different fermentation conditions using Minitab^®^ statistical software, version 20.3 (Minitab Inc., State College, PA, USA). Graphs were generated using SigmaPlot^®^ software, version 15 (Grafiti LLC, Palo Alto, CA, USA).

Volatile compounds were compared across conditions using one-way ANOVA and Tukey’s test (*p* < 0.05) with Minitab^®^ v. 20.3 (Minitab Inc., State College, PA, USA). To discriminate key compounds, the volatilome of unfermented M2 medium was compared with that of the samples from different fermentation times (6, 17.5, and 24 h). Multivariate analyses were also performed to identify the volatile compounds that contributed most across conditions. The MetaboAnalyst 6.0 platform (https://www.metaboanalyst.ca/, accessed on 24 March 2026) was used to generate a heat map using hierarchical clustering and principal component analysis (PCA). Before this, the data were normalized by summation, transformed using the cube root, and scaled by autoscaling (centered on the mean and divided by the standard deviation of each variable).

## 3. Results

### 3.1. Monocultures and Co-Cultures

**Figure 2:**
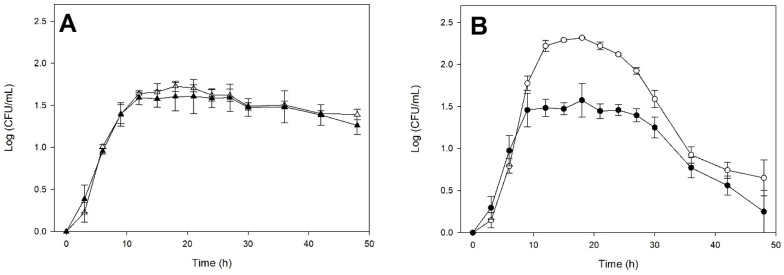

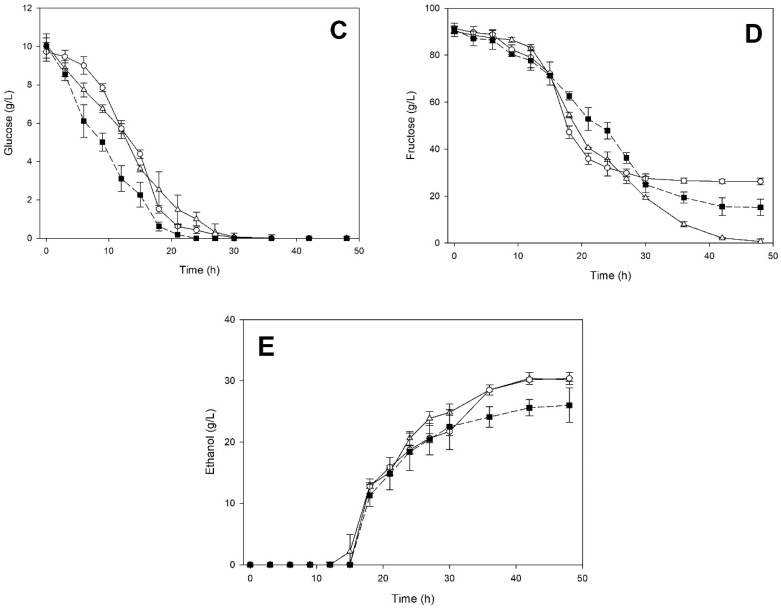
Fermentation kinetics of *Kluyveromyces marxianus* ITD-01005 and *Saccharomyces cerevisiae* ITD-00185 in monoculture and co-culture in M2 medium. Cell-density of *S. cerevisiae* (**A**) in monoculture (△) and in co-culture (▲). Cell density of *K. marxianus* (**B**) in monoculture (○) and in co-culture (●). Glucose (**C**), fructose (**D**), and ethanol (**E**) concentrations measured in the culture supernatant for the *K. marxianus* monoculture (○), the *S. cerevisiae* monoculture (△), and the co-culture (■). Data points represent the average of three biological replicates. Error bars indicate standard deviations.

Figure 2 shows the fermentation kinetics (biomass, glucose, fructose, and ethanol) of *S. cerevisiae* and *K. marxianus* in monoculture and co-culture. The growth kinetics of *S. cerevisiae* (Figure 2A) exhibit almost identical behavior in both culture types. In contrast, Figure 2B shows that *K. marxianus* coexists without problems during the first 6 h of incubation in co-culture with *S. cerevisiae*. However, these first hours of co-culture triggered a response in *S. cerevisiae* that significantly affects the viability of *K. marxianus* from 9 h onward. The *K. marxianus* monoculture exhibited a brief stationary phase before reaching a maximum biomass production of 2.27 log CFU/mL, which was significantly higher (*p* < 0.05) than that of *S. cerevisiae* (1.70 log CFU/mL). Despite a decrease in its viability compared to monoculture, *K. marxianus* exhibited a longer stationary phase of approximately 18 h in co-culture.

Regarding sugar consumption, a rapid depletion of glucose was observed (Figure 2C). Glucose was consumed similarly by both monocultures, but the co-culture showed slightly faster consumption. Figure 2D shows similar fructose consumption by the three cultures, with slow consumption during the first 15 h of incubation. The rate of fructose consumption accelerated from 15 to 30 h, showing complete depletion only in the *S. cerevisiae* monoculture. In the case of the *K. marxianus* monoculture and the co-culture, fructose consumption plateaued, becoming very slow after 30 h of incubation. It is probably related to the pronounced decline in *K. marxianus* viability during the death phase, both in monoculture and in co-culture.

Figure 2E shows that ethanol production began after 12 to 15 h of incubation in all cultures. Production was very similar in both monocultures and slightly lower in co-cultures. At the end of fermentation, the *S. cerevisiae* and *K. marxianus* monocultures reached comparable concentrations (30.19 ± 0.27 g/L and 30.40 ± 0.98 g/L, respectively) while the final ethanol concentration in co-culture was 26.03 ± 2.82 g/L, showing a significant reduction compared to monoculture.

### 3.2. Cultures for Indirect Interaction Assays

**Figure 3:**
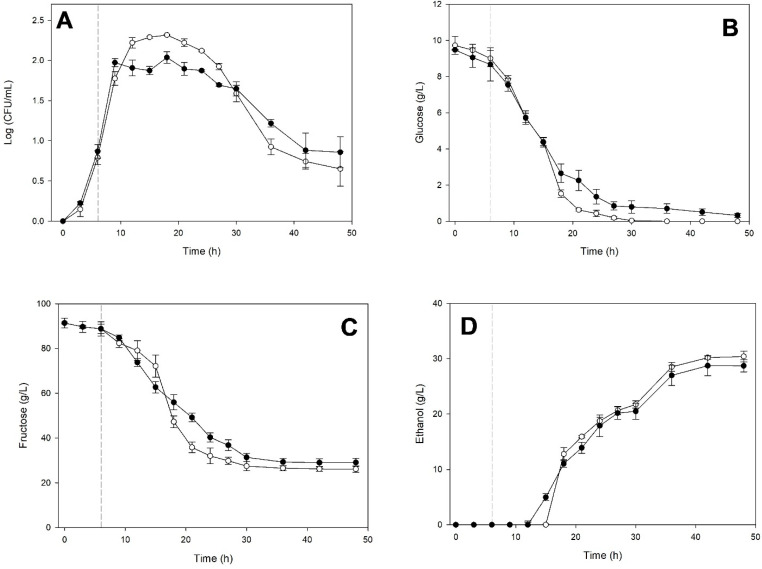
Fermentation kinetics of *Kluyveromyces marxianus* ITD-01005 in monoculture (○) and on the cell-free medium (●) previously fermented by *Saccharomyces cerevisiae* ITD-00185 for 6 h. Cell-density (**A**) and glucose (**B**), fructose (**C**), and ethanol (**D**) concentrations. Data points represent the average of three biological replicates. Error bars indicate standard deviations. A vertical dashed line indicates the incubation time during which medium exchange was performed.

Figure 3 shows the fermentation kinetics of *K. marxianus* in monoculture and when cultured on the medium in which *S. cerevisiae* had previously grown for 6 h.

The growth kinetics shown in Figure 3A indicate that when *S. cerevisiae* entered the exponential phase, it produced extracellular compounds that slightly inhibited *K. marxianus*. It tolerated this medium for only 3 h, as its growth was like that in monoculture until 9 h of incubation. After this time, it entered a stationary phase lasting approximately 15 h, followed by a death phase like that observed in its monoculture. Glucose (Figure 3B) and fructose (Figure 3C) consumption indicate similar behaviors. It should be noted that *K. marxianus* did not consume all the glucose when cultured on medium previously fermented by *S. cerevisiae*. In this medium, ethanol production (Figure 3D) started three hours earlier than in the *K. marxianus* monoculture. Still, it then showed a similar behavior to the latter.

**Figure 4:**
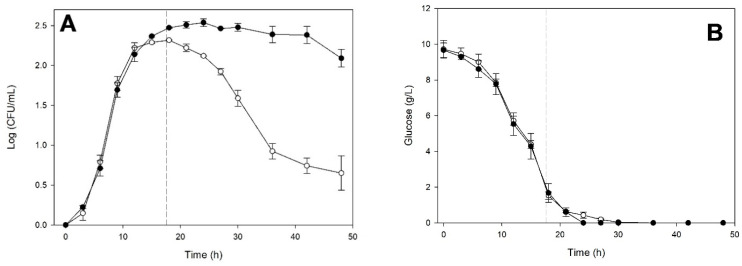

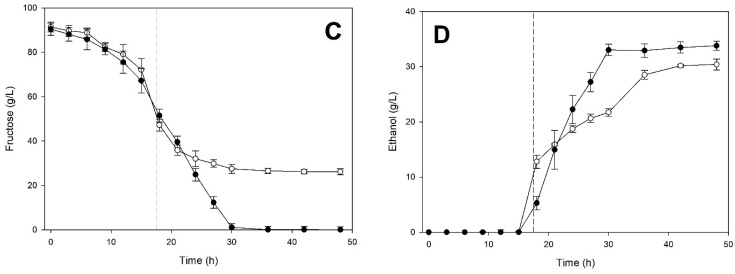
Fermentation kinetics of *Kluyveromyces marxianus* ITD-01005 in monoculture (○) and on the cell-free medium (●) previously fermented by *Saccharomyces cerevisiae* ITD-00185 for 17.5 h. Cell-density (**A**), glucose (**B**), fructose (**C**), and ethanol (**D**) concentrations. Data points represent the average of three biological replicates. Error bars indicate standard deviations. A vertical dashed line indicates the incubation time during which medium exchange was performed.

Conversely, when *S. cerevisiae* reached the stationary phase, it left a fermented medium with a composition that significantly enhanced the fermentation kinetics of *K. marxianus* (Figure 4).

The growth kinetics shown in Figure 4A indicate that *K. marxianus* growth was stimulated, resulting in a prolonged stationary phase of approximately 24 h. In contrast, the monoculture was already in the death phase. Total glucose consumption was observed, like for monoculture (Figure 4B). It is noteworthy that the culture on the medium previously fermented by *S. cerevisiae* achieved total fructose consumption (Figure 4C), unlike the monoculture, which exhibited abundant residual fructose (30 g/L). Figure 4D shows that ethanol production began at 15 h in both cases. Still, the *K. marxianus* culture in the medium previously fermented by *S. cerevisiae* produced significantly more ethanol (*p* < 0.05), which ceased when fructose was depleted.

**Figure 5:**
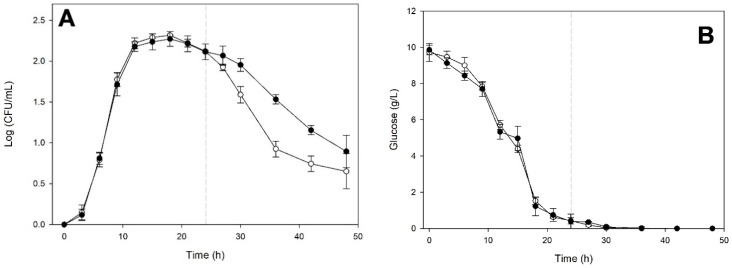

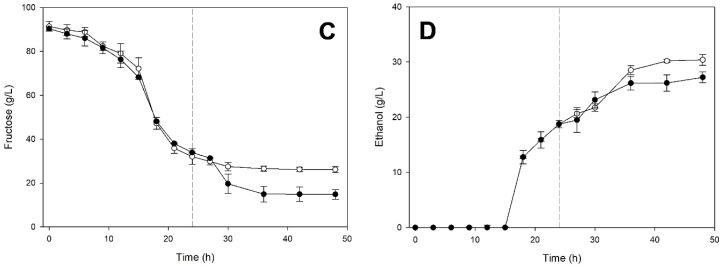
Fermentation kinetics of *Kluyveromyces marxianus* ITD-01005 in monoculture (○) and on the cell-free medium (●) previously fermented by *Saccharomyces cerevisiae* ITD-00185 for 24 h. Cell-density (**A**), glucose (**B**), fructose (**C**), and ethanol (**D**) concentrations. Data points represent the average of three biological replicates. Error bars indicate standard deviations. A vertical dashed line indicates the incubation time during which medium exchange was performed.

The cultivation of *K. marxianus* in the medium previously fermented by *S. cerevisiae* until the beginning of the death phase is shown in Figure 5.

The *K. marxianus* culture had already begun its death phase when it was transferred to M2 medium previously fermented by *S. cerevisiae* for 24 h (Figure 5A). However, this figure shows that the change slowed the progression of the death phase in *K. marxianus*. Glucose was almost depleted when the *K. marxianus* culture was transferred, and no differences were observed compared to monoculture (Figure 5B). Still, fructose consumption was prolonged by an additional 6–9 h (Figure 5C). Figure 5D shows that ethanol production was not affected by the transfer of the K. marxianus culture to the medium previously fermented by *S. cerevisiae*.

**Figure 6:**
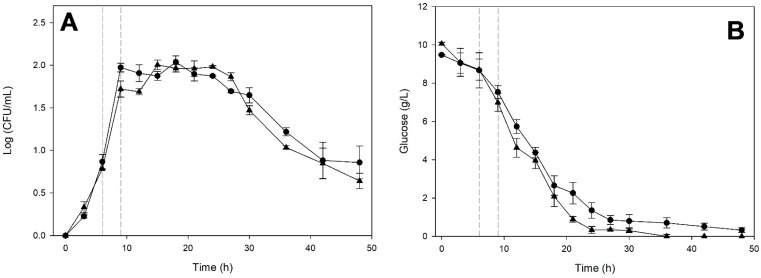

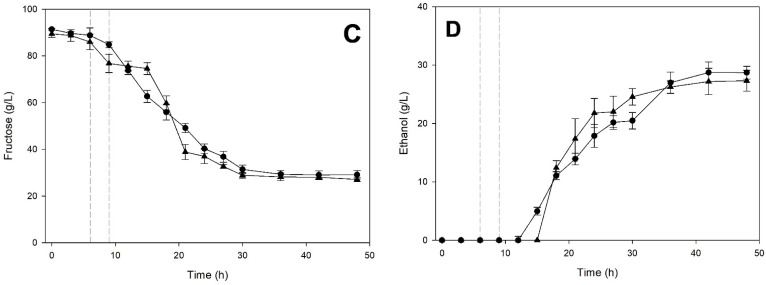
Fermentation kinetics of *Kluyveromyces marxianus* ITD-01005 growing on the cell-free media previously fermented for 6 h (●) by *Saccharomyces cerevisiae* ITD-00185 and in a cross-exposure assay (▲) from 6 to 9 h. Cell-density (**A**) and glucose (**B**), fructose (**C**), and ethanol (**D**) concentrations. Data points represent the average of three biological replicates. Error bars indicate standard deviations. The two vertical dashed lines indicate the incubation times during which media exchange was performed.

**Figure 7:**
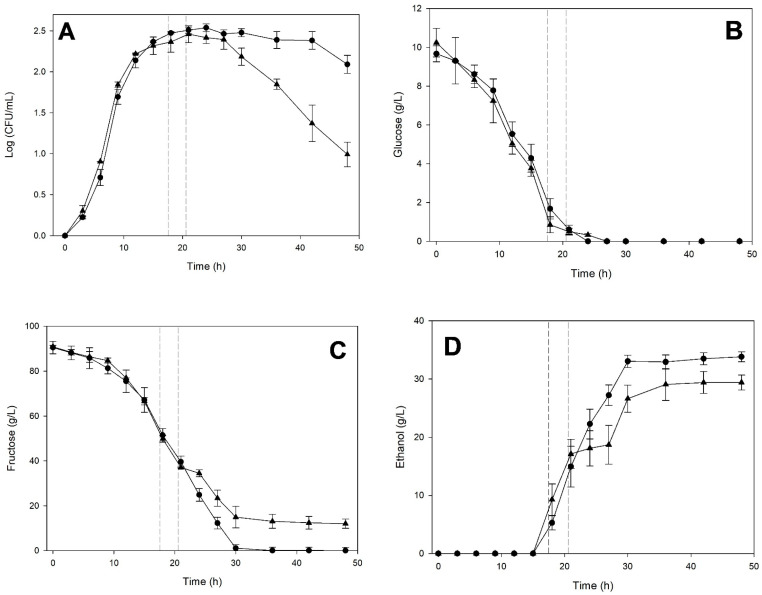
Fermentation kinetics of *Kluyveromyces marxianus* ITD-01005 growing on the cell-free media previously fermented for 17.5 h (●) by *Saccharomyces cerevisiae* ITD-00185 and in a cross-exposure assay (▲) from 17.5 to 20.5 h. Cell-density (**A**) and glucose (**B**), fructose (**C**), and ethanol (**D**) concentrations. Data points represent the average of three biological replicates. Error bars indicate standard deviations. The two vertical dashed lines indicate the incubation times during which media exchange was performed.

**Figure 8:**
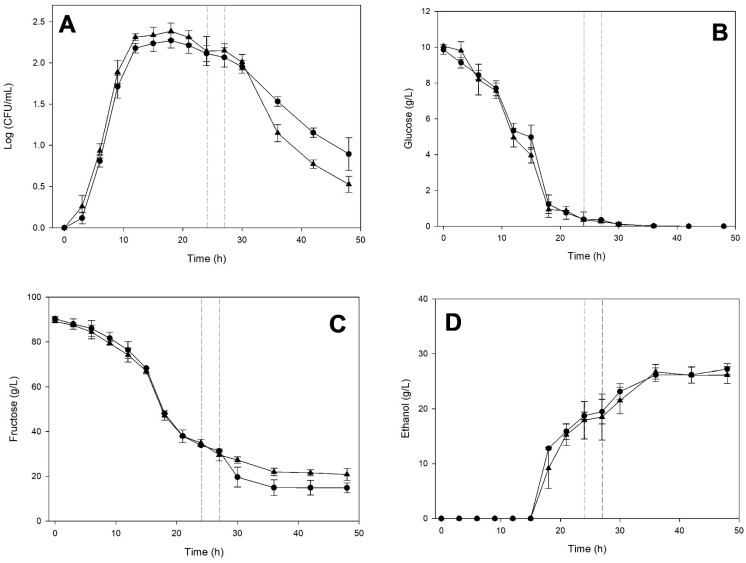
Fermentation kinetics of *Kluyveromyces marxianus* ITD-01005 growing on the cell-free media previously fermented for 24 h (●) by *Saccharomyces cerevisiae* ITD-00185 and in a cross-exposure assay (▲) from 24 to 27 h. Cell-density (**A**) and glucose (**B**), fructose (**C**), and ethanol (**D**) concentrations. Data points represent the average of three biological replicates. Error bars indicate standard deviations. The two vertical dashed lines indicate the incubation times during which media exchange was performed.

Regarding the cross-exposure assays, results are shown in Figure 6, Figure 7 and Figure 8. The effect of the cross-exposure assay from 6 to 9 h of incubation was not significantly different from that caused by the simple exchange at 6 h (exponential phase), beyond stimulating a late (36 h) depletion of glucose and an advance of 3 h in the start of ethanol production (Figure 6). The cross-exposure assay from 17.5 to 20.5 h of incubation showed a more pronounced effect than the simple exchange at 24 h (stationary phase), as shown in Figure 7.

This cross-exposure assay did not prolong the stationary phase, but the death phase was slower than in the monoculture. Glucose was completely consumed, but fructose was incompletely consumed, resulting in a residual concentration of approximately 10 g/L. Similarly, slightly lower ethanol production was observed compared to the single exchange. Furthermore, Figure 8 shows that there were no significant differences between the cross-exposure assay from 24 to 27 h and the single exchange at 24 h (death phase).

Some fermentation parameters were calculated to complete the kinetic analysis of cultures behavior (Table 1). Significant differences were found in some $\mu_{max}$ values. The *K. marxianus* monoculture showed the highest value, while *S. cerevisiae* in the co-culture showed the lowest. In general, the difference is minimal between the $\mu_{max}$ values for the *K. marxianus* monoculture and those corresponding to the various exchanges tested. When comparing monocultures with co-culture, *S. cerevisiae* shows a slight decrease in $\mu_{max}$ (15%), which is not statistically significant (*p* < 0.05). Conversely, the $\mu_{max}$ of *K. marxianus* in co-culture decreased by 27% (*p* < 0.05) compared to its monoculture counterpart. No significant differences (*p* < 0.05) were observed between the $\mu_{max}$ values of the various cultures with single or cross exchanges.

Table 1 also shows that the shortest lag time was observed in *S. cerevisiae* in co-culture. At the same time, *K. marxianus* monoculture and single exchange at 24 h had the longest lag time. The $t_{lag}$ of *K. marxianus* in co-culture decreased by 46% (*p* < 0.05) compared to its monoculture counterpart. Regarding ethanol yield, *K. marxianus* monoculture showed the highest value. It was almost 25% higher (*p* < 0.05) than that of co-culture and *S. cerevisiae* monoculture, which showed the lowest values.

### 3.3. Composition of the Cell-Free Extracts of Media Fermented by S. cerevisiae

**Figure 9:**
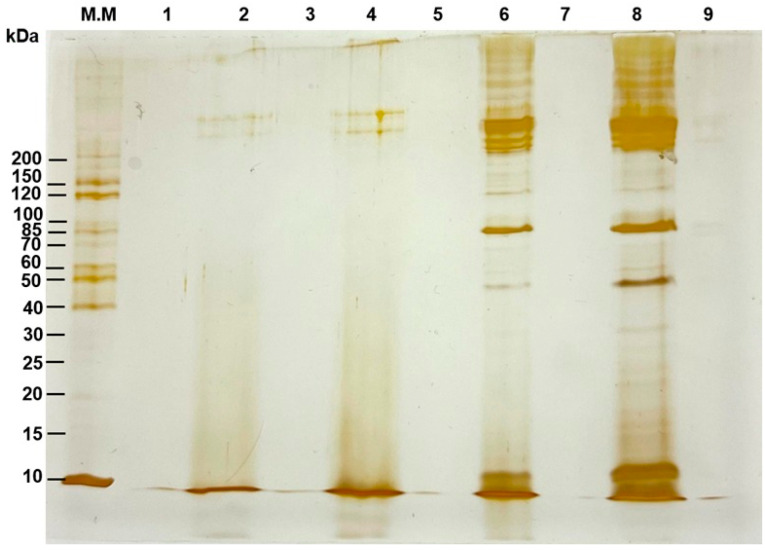
Protein profiles of *Saccharomyces cerevisiae* cell-free supernatants resolved by silver-stained SDS–PAGE (15% polyacrylamide/bisacrylamide gel). Line M.M., molecular weight marker; Line 1, empty; Line 2, unfermented M2 medium; Line 3, empty; Line 4, M2 medium fermented for 6 h by *S. cerevisiae*; Line 5, empty; Line 6, M2 medium fermented for 17.5 h by *S. cerevisiae*; Line 7, empty; Line 8, M2 medium fermented for 24 h by *S. cerevisiae*.

The denaturing SDS-PAGE gel shown in Figure 9 allows visualization of differences in the protein profile of cell-free *S. cerevisiae* supernatants as a function of fermentation time. The sample from 6 h of fermentation shows no significant differences compared to the unfermented M2 medium. In contrast, the samples from 17.5 and 24 h of incubation showed intense signals of high molecular weight (≥200 kDa) and well-defined bands around ~85 and ~50 kDa, in addition to intense signals in the ~10–15 kDa range. The 15% gel was unable to adequately resolve species with apparent mobility below 10 kDa, which are the peptides of interest.

**Figure 10:**
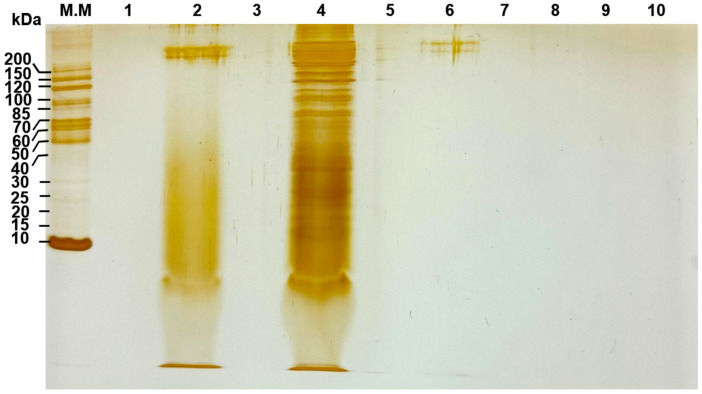
Protein profiles of *Saccharomyces cerevisiae* cell-free supernatants resolved by silver-stained SDS–PAGE (20% polyacrylamide/bisacrylamide gel). Line M.M., molecular weight marker; Line 1, empty; Linne 2, unfermented M2 medium; Line 3, empty; Line 4, M2 medium fermented for 6 h by *S. cerevisiae*.

Thus, the protein precipitates of the unfermented M2 medium and the 6 h fermentation samples were run on a 20% gel (Figure 10). It enabled better retention and greater definition at the lower end of the gel, improving visualization of the low-molecular-weight fraction. This figure shows a clear difference in the protein profile of the 6 h fermentation sample compared to the unfermented M2 medium. The 6 h fermentation sample showed a diffuse signal in the lower region of the gel, without any band clearly distinguishable from the unfermented medium in the <10 kDa range.

**Figure 11:**
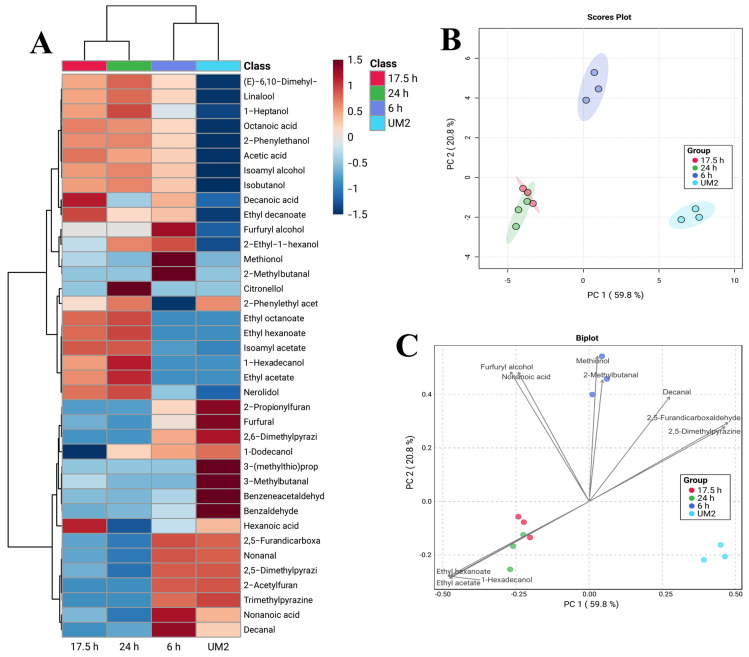
Analysis of the volatilome in unfermented M2 medium (UM2) and M2 medium fermented by *S. cerevisiae* for 6, 17.5, and 24 h. (**A**) Hierarchical cluster heatmap. (**B**) PCA scores plot. (**C**) PCA biplot.

Figure 11 exhibits the results of the volatile compounds detected in cell-free extracts of medium M2 fermented by *S. cerevisiae* for 6, 17.5, and 24 h.

The heat map (Figure 11A) shows a hierarchical clustering of the volatilome from the 6 h fermentation supernatant with that of the unfermented M2 medium. Similarly, the 17.5 h and 24 h fermentations formed a separate volatilome cluster. Hexanoic, octanoic, and decanoic acids, along with 2-phenylethanol, isoamyl alcohol, methionol, and isobutanol, were found primarily in the 17.5 h and 24 h fermentations. The unfermented medium (UM2) showed higher concentrations of compounds, including furfural and some pyrazines. The 6 h fermentation samples represented a transitional state, showing the appearance of several compounds at considerably lower concentrations than those observed at 17.5 h and 24 h fermentation.

One-way analysis of variance followed by Tukey’s test (Supplementary Table S1) showed that *S. cerevisiae* significantly altered the volatile compound profile of medium M2. The greatest differences between conditions were concentrated in organic acids and several higher alcohols. Specifically, among the organic acids, the most notable changes were observed in hexanoic acid, octanoic acid, and decanoic acid. Hexanoic acid showed the lowest concentration at 6 h (0.0365 ± 0.0188 mg/L), reached its maximum value at 17.5 h (1.1070 ± 0.1144 mg/L), and decreased at 24 h (0.4450 ± 0.3863 mg/L), showing significant differences (*p* < 0.05) between 17.5 h and the other fermentation times. Octanoic acid showed the most marked pattern, with a lower concentration at 6 h (0.7411 ± 0.3564 mg/L) and significantly higher concentrations (*p* < 0.05) at 17.5 h (16.7968 ± 2.4606 mg/L) and 24 h (13.9375 ± 2.8828 mg/L). Decanoic acid followed a similar trend, with a slight increase at 6 h (0.4827 ± 0.2439 mg/L), a maximum at 17.5 h (13.3535 ± 2.4586 mg/L), and a decrease at 24 h (3.7649 ± 0.4817 mg/L).

The alcohol group showed significant increases in some compounds. 2-Phenylethanol appeared at 6 h of fermentation (1.7746 ± 0.4126 mg/L) and increased markedly at 17.5 h (53.0510 ± 5.0369 mg/L) and 24 h (48.8296 ± 6.2011 mg/L), with no significant difference (*p* > 0.05) between these last two fermentation times. Similarly, isoamyl alcohol showed a similar pattern, increasing from 2.9785 ± 2.5796 mg/L (6 h fermentation) to 24.9851 ± 1.3463 mg/L (17.5 h fermentation) and 24.3695 ± 3.8692 mg/L (24 h fermentation).

Principal component analysis (PCA) of volatile compounds showed that the unfermented medium (UM2) was markedly distinct from the fermented samples (Figure 11B). It also shows that the 6 h fermentation formed an independent group, indicating a transitional volatile profile compared to the unfermented medium and the more advanced fermentations, which clustered together. The first two principal components explained 80.6% of the total variation, with PC1 accounting for 59.8% and PC2 for 20.8%. The PCA biplot (Figure 11C) showed that the discrimination between conditions was primarily associated with a small set of compounds that contributed significantly to the first two components. The 17.5 h and 24 h samples were mainly associated with ethyl acetate, ethyl hexanoate, and 1-hexadecanol. In contrast, the 6 h samples were associated with methionol and 2-methylbutanal.

## 4. Discussion

Byrne et al. [22] reported the use of media-exchange experiments to determine the inhibition of cell growth by starvation for leucine in *S. cerevisiae*. However, they used only one strain, which they transferred, by centrifugation and cell washing, from a medium containing leucine to one without leucine. To our knowledge, there are no reports of experiments exchanging yeast cells between their partially fermented media. This strategy was implemented because several previous studies reported co-cultures avoiding cell–cell contact [2,3,5,23,24,25,26] by membranes or porous cartridges to separate the cells. However, each yeast cell was continuously exposed to the metabolites of the other strain. In the approach proposed here, we sought to determine whether *S. cerevisiae* constitutively produces compounds that inhibit the growth of *K. marxianus*, or whether this inhibition requires prior physical or chemical contact.

The decrease in *K. marxianus* viability in co-culture relative to monoculture can indicate a metabolic/physiological adaptation in *S. cerevisiae* that induces biotic stress on *K. marxianus*. However, *K. marxianus* must also have made its own adjustments that allowed it to exhibit a long stationary phase. It agrees with the findings of Luyt et al. [26], who used synthetic grape must as the medium for their experiments, and with those of López et al. [2], who used M11 medium (designed to resemble the agave juice used to produce mezcal or tequila in Mexico). They reported that co-culture of *S. cerevisiae* and *K. marxianus* with physical contact significantly reduced *K. marxianus* viability. Furthermore, the higher biomass production of *K. marxianus* agrees with that reported by López et al. [2], who found that *K. marxianus* DU3 (isolated from artisanal mezcal fermentation) produces twice as much biomass as *S. cerevisiae*. They also reported that, in mixed fermentations with direct contact, *S. cerevisiae* showed a clear competitive advantage over *K. marxianus*, particularly after 24 h, when *S. cerevisiae* came to dominate the culture.

However, the response to co-culture is not uniform since phenotypic variation has been observed among *K. marxianus* strains in the presence of *S. cerevisiae*. In the work of Nolasco-Cancino et al. [4], the comparison between monocultures and co-cultures suggested an apparent negative effect of *S. cerevisiae* on *K. marxianus*. Conversely, the *K. marxianus* PA16 strain shows greater tolerance to co-culture, indicating a strain-dependent behavior with respect to *S. cerevisiae*. However, it is important to note that the matrix evaluated was agave juice with bagasse, and that the cell count in the co-cultures corresponds to the total population of both species, thus precluding the discrimination of the specific effect on the viability of each yeast.

The results shown here appear unusual, as *K. marxianus* strains isolated from agave juice fermentation have demonstrated a high fermentative capacity, with a predominance in this type of must. It has been explained by their ability to grow on fructans, due to their fructanase activity [27].

The slight decrease in $\mu_{max}$ for *S. cerevisiae* in coculture is quantitative evidence of the impact of competing with another high-fermenting yeast, such as *K. marxianus*, in a nutrient-limited culture medium. However, it is the latter yeast that is more significantly impacted by competition in co-culture. It is consistent with the more abundant growth of *K. marxianus* in monoculture, as shown in Figure 2. $t_{lag}$ data show that *S. cerevisiae* activates its growth more rapidly in co-culture than in monoculture. At the same time, *K. marxianus* takes longer to adapt to the competitive conditions of coculture. These two parameters allow comparison of initial fermentation times, while ethanol yield helps compare the completion of the cultures.

The observed ethanol production does not agree with that reported by Nolasco-Cancino et al. [4], who found that ethanol production from pure cultures of *S. cerevisiae* (21.78 g/L) and *P. kudriavzevii* (11.63 g/L) increased when each strain was combined with *K. marxianus*. It is noteworthy that ethanol production began late despite significant sugar consumption from the start of fermentation. Furthermore, the onset of ethanol production coincides with high cell density, which consumes all the oxygen entering the culture medium due to agitation. Once dissolved oxygen became insufficient, the cell’s metabolism shifted from respiratory to respiro-fermentative. It can explain why cell growth ceased while sugar consumption continued and ethanol production was observed.

It is noteworthy that this *K. marxianus* strain exhibits the highest ethanol yield (0.397 ± 0.001 g/g), representing almost 78% of the maximum theoretical yield (0.51 g/g) for alcoholic fermentation. It is believed that ethanol accumulation induces environmental stress, thereby eliminating sensitive species [28,29]. However, this is not the case for the *K. marxianus* strain used, as its high production in monoculture (Figure 1) demonstrates that it tolerates ethanol well, even exhibiting the highest yield. It is also striking that *S. cerevisiae* had the lowest ethanol yield, despite its reputation for high ethanol production capacity. It is further reflected in the coculture, which also shows a very low value, providing additional evidence that *S. cerevisiae* negatively affects the development of *K. marxianus* and dominates this fermentation.

The composition of the medium partially fermented by *S. cerevisiae* for 6 h exerted a clear inhibition of *K. marxianus* growth, although not as marked as that resulting from co-culture. It suggests that *S. cerevisiae* would release inhibitory compounds during exponential growth, even without physical contact with *K. marxianus* or its metabolites in the culture medium. Branco et al. [30] demonstrated that certain strains of *S. cerevisiae* are capable of producing antimicrobial peptides with broad-spectrum fungicidal activity, including against *K. marxianus*.

An intense signal near the running front (10 kDa) was observed in both the unfermented medium and the fermented supernatants. It is consistent with the contribution of casein peptone to M2 medium. It provides a mixture of low-molecular-weight peptides and generates a high background signal in that region, making it difficult to discriminate specific extracellular peptides produced by *S. cerevisiae*. Thus, the results of the protein analysis are inconclusive; they do not allow us to affirm or deny the presence of low-molecular-weight peptides after 6 h of fermentation by *S. cerevisiae*. These peptides may be present at concentrations below the assay’s detection limit. However, the observed inhibition could be due to the combined action of low-concentration molecules, such as peptides, organic acids, and alcohols.

Some organic acids have been reported to decrease the specific growth rate and biomass yield and to prolong the lag phase in some non-Saccharomyces yeasts. Legras et al. [31] indicated that the toxicity of octanoic acid and decanoic acid increases in the presence of ethanol and at low pH. These conditions favor their entry into the cell in undissociated form and subsequent intracellular dissociation, resulting in a decrease in intracellular pH. Furthermore, due to their lipophilic nature, these acids also affect the cell membrane, especially decanoic acid, and trigger responses associated with oxidative stress. Consistently, Pohl et al. [32] indicated in their review that the main mechanism of action of fatty acids involves disruption of the cell membrane, with increased fluidity, loss of integrity, and leakage of intracellular components.

Likewise, Chen and Fink [33] determined that 2-phenylethanol inhibited germ tube and biofilm formation in *Candida albicans*. Hua et al. (2014) [34] showed that this compound inhibits spore germination and growth of *Aspergillus flavus*. Liu et al. [8] pointed out the antifungal activity of 2-phenylethanol against *Penicillium italicum* and *P. digitatum*. They proposed that this aromatic alcohol inhibits fungal growth by repressing pathways involved in amino acid and protein biosynthesis, DNA replication, and the cell cycle, while activating processes associated with autophagy and the stress response.

The analysis of peptides <10 kDa is performed using systems optimized for low molecular weight (e.g., Tris-trycin SDS-PAGE). However, the system used in this study (Laemmli-type SDS-PAGE) shows a diffuse signal indicating the presence of at least protein fragments. It therefore cannot be considered conclusive regarding the presence or absence of low molecular weight peptides produced by *S. cerevisiae* after 6 h of fermentation in M2 medium. A conclusive study will require the use of direct mass spectrometry (MALDI-TOF) or mass spectrometry coupled to liquid chromatography (LC-MS/MS).

Conversely, when *S. cerevisiae* was grown for longer periods (17.5 and 24 h), its cell-free media supported the growth of *K. marxianus*, promoting greater growth, prolonging the stationary phase compared to its monoculture, depleting the available sugars, and producing more ethanol. It can be explained as a recycling phenomenon of residual compounds from *S. cerevisiae* metabolism or autolysis during its stationary phase. It would release compounds such as amino acids, vitamins, nitrogenous bases, and other metabolites. The results of the protein analysis confirm the abundant presence of proteins of diverse molecular weights in the cell-free extracts fermented for 17.5 and 24 h (Figure 9).

The cross-exposure assay during the death phase did not yield results different from those of simple exchange at that stage of the culture, even in the *K. marxianus* monoculture. Therefore, by that time, the *K. marxianus* culture could have progressed so far that it could no longer be significantly affected. Collectively, the results of the present study demonstrate that these findings are independent of the use of single- or cross-exchange culture media.

In substrate-limited media, like M2 medium, microbial interactions are complex, including both positive (protocooperation or synergism, commensalism) and negative (antagonism, competition) interactions, which affect substrate consumption and product formation [29,35]. The premature decline of non-*Saccharomyces* populations is multifactorial. It can include competition for nutrients such as sugars and oxygen, production of inhibitory metabolites, and even competition for space [29]. The relevance of abiotic factors such as the composition of the culture medium has been highlighted [35]. Our research group has demonstrated it for strains derived from agave fermentation [36]. That previous study showed that a nutritionally rich medium can improve the survival of non-*Saccharomyces* yeasts. However, negative interactions within mixed microbial populations should not be considered detrimental to the final product. Amensalism and antagonism have been observed to reduce fermentation time and improve aroma complexity in spirits [28]. Microbial interactions (direct cell–cell contact or indirect metabolite mediation) can modulate microbial growth and activity, as well as drive ongoing environmental changes [29]. These authors conclude that studying these interactions will enhance the understanding of fermentations and the development of production methods to obtain programmed flavor profiles in alcoholic beverages.

## 5. Conclusions

The effect of compounds excreted by *S. cerevisiae* ITD-00185 into the culture medium on the growth and fermentative capacity of *K. marxianus* ITD-01005 depends on the growth phase of the culture. During the exponential phase, cell–cell contact is responsible for most of the inhibition of *K. marxianus* growth. However, *S. cerevisiae* monoculture produces extracellular compounds during the exponential phase that also contribute to the inhibition of *K. marxianus*. In contrast, during the maintenance phase and the early death phase, the physiology and metabolism of *S. cerevisiae* changed appreciably, releasing proteins and likely other metabolites, such as amino acids, vitamins, nitrogenous bases, and others. These compounds were nutrients that stimulated the development of *K. marxianus*, slowing and prolonging its death phase.

## Figures and Tables

**Table 1:** Fermentation parameters of the cultures performed in M2 medium. $\mu_{max}$ and $t_{lag}$ were calculated from Gompertz Equation fits. Plots of the fits are shown in Supplementary Figures S1–S10. These figures show the corresponding R^2^ values, which range from 0.9678 to 0.9996.

Culture		$\mu_{max}$ (h^−1^)	$t_{lag}$ (h)	*Y_P/S_* (g/g)
Monoculture	Sc	0.26 ± 0.01 ^de^	2.16 ± 0.13 ^cde^	0.307 ± 0.002 ^d^
	Km	0.37 ± 0.03 ^a^	3.77 ± 0.37 ^a^	0.397 ± 0.001 ^a^
Coculture	Sc	0.22 ± 0.03 ^e^	1.39 ± 0.67 ^e^	0.298 ± 0.034 ^d^
	Km	0.27 ± 0.02 ^cde^	2.02 ± 0.21 ^de^	
Simple exchange	6 h	0.33 ± 0.03 ^abc^	2.94 ± 0.08 ^abc^	0.396 ± 0.031 ^ab^
	17.5 h	0.30 ± 0.02 ^bcd^	3.34 ± 0.01 ^ab^	0.334 ± 0.004 ^bcd^
	24 h	0.35 ± 0.02 ^ab^	3.65 ± 0.12 ^a^	0.311 ± 0.021 ^d^
Cross-exposure assays	6 h	0.25 ± 0.00 ^de^	2.24 ± 0.04 ^cde^	0.375 ± 0.022 ^abc^
	17.5 h	0.31 ± 0.02 ^bcd^	2.71 ± 0.18 ^bcd^	0.331 ± 0.027 ^cd^
	24 h	0.34 ± 0.02 ^ab^	3.00 ± 0.51 ^abc^	0.339 ± 0.024 ^abcd^

The data are averages of triplicates ± standard deviation. Different letters mean significant difference (*p* < 0.05) among values in the same column. Sc: *S. cerevisiae*, Km: *K. marxianus*.

## Data Availability

The original contributions presented in this study are included in the article/Supplementary Materials. Further inquiries can be directed to the corresponding author.

## Supplementary Materials

The following supporting information can be downloaded at: https://www.mdpi.com/article/10.3390/microorganisms14040890/s1, Figure S1: Fitting the Gompertz Equation (dotted line) to the growth data of the S. cerevisiae monoculture. Figure S2: Fitting the Gompertz Equation (dotted line) to the growth data of the *K. marxianus* monoculture. Figure S3: Fitting the Gompertz Equation (dotted line) to the growth data of the *S. cerevisiae* in coculture. Figure S4: Fitting the Gompertz Equation (dotted line) to the growth data of the *K. marxianus* in coculture. Figure S5: Fitting the Gompertz Equation (dotted line) to the growth data of the *K. marxianus* on the cell-free medium previously fermented by *Saccharomyces cerevisiae* for 6 h. Figure S6: Fitting the Gompertz Equation (dotted line) to the growth data of the *K. marxianus* on the cell-free medium previously fermented by *Saccharomyces cerevisiae* for 17.5 h. Figure S7: Fitting the Gompertz Equation (dotted line) to the growth data of the *K. marxianus* on the cell-free medium previously fermented by *Saccharomyces cerevisiae* for 24 h. Figure S8: Fitting the Gompertz Equation (dotted line) to the growth data of the *K. marxianus* in a cross-exposure assay from 6 to 9 h. Figure S9: Fitting the Gompertz Equation (dotted line) to the growth data of the *K. marxianus* in a cross-exposure assay from 17.5 to 20.5 h. Figure S10: Fitting the Gompertz Equation (dotted line) to the growth data of the *K. marxianus* in a cross-exposure assay from 24 to 27 h. Table S1: Semi-quantification of volatile compounds (mg/L). Data are presented as mean ± standard deviation of triplicate determinations. Different letters within the same row indicate significant differences among fermentation times (one-way ANOVA followed by Tukey’s test, *p* < 0.05). RI_AMDIS: retention index of each compound determined using AMDIS software under the same analytical conditions. RI_L: retention index of each compound previously reported in the literature (* indicates retention index obtained using a DB-Wax column). ND: not detected. UM2: unfermented M2 medium; 6 h: M2 medium fermented by *S. cerevisiae* for 6 h; 17.5 h: M2 medium fermented by *S. cerevisiae* for 17.5 h; 24 h: M2 medium fermented by *S. cerevisiae* for 24 h.
