# Supplementary material for: Extracellular Metabolites from Saccharomyces cerevisiae Modulate the Growth and Fermentative Performance of Kluyveromyces marxianus
Source: Microorganisms. 2026 Apr 16;14(4):890. doi: 10.3390/microorganisms14040890 (PMC13118812; doi:10.3390/microorganisms14040890)
Supplement: Supplementary file 1 [file microorganisms-14-00890-s001.zip › Suplementary_Figures_S1_S10.pdf]

microorganisms

# Extracellular metabolites from *Saccharomyces cerevisiae* modulate the growth and fermentative performance of *Kluyveromyces marxianus*

**Jairo Gallardo-Rivera, Oscar E. Soto-Malpica, Erick D. Acosta-García, Perla G. Vázquez-Ortega, Juan A. Rojas-Contreras and Nicolas O. Soto-Cruz ***

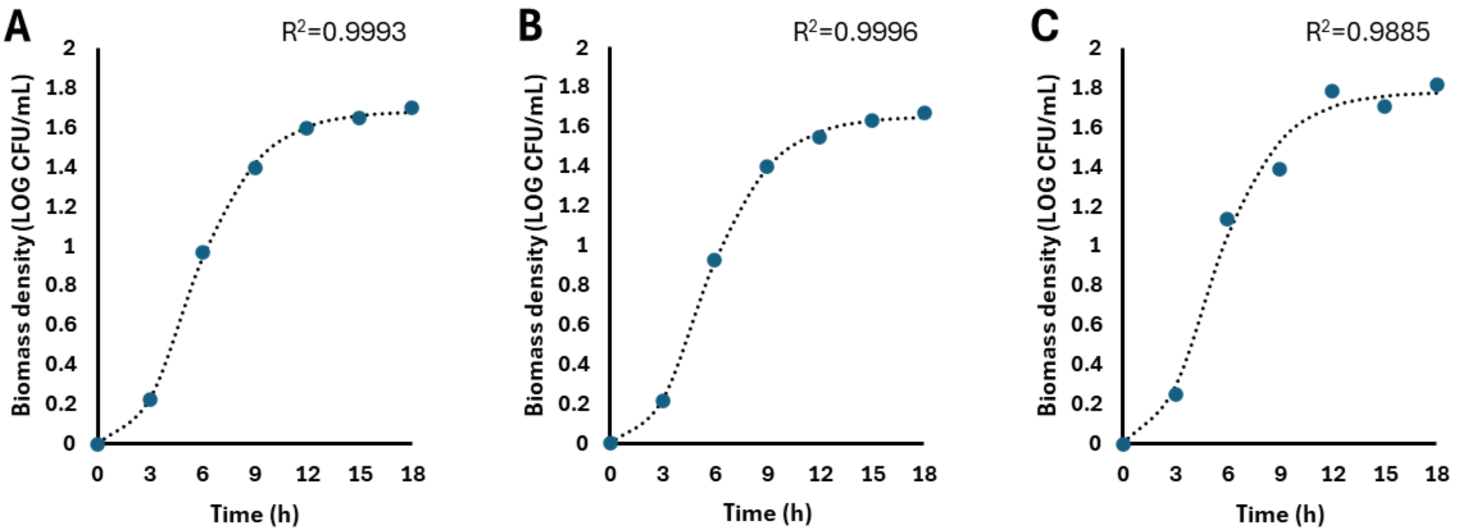


**Figure S1.** Fitting the Gompertz Equation (dotted line) to the growth data of the *S. cerevisiae* monoculture.

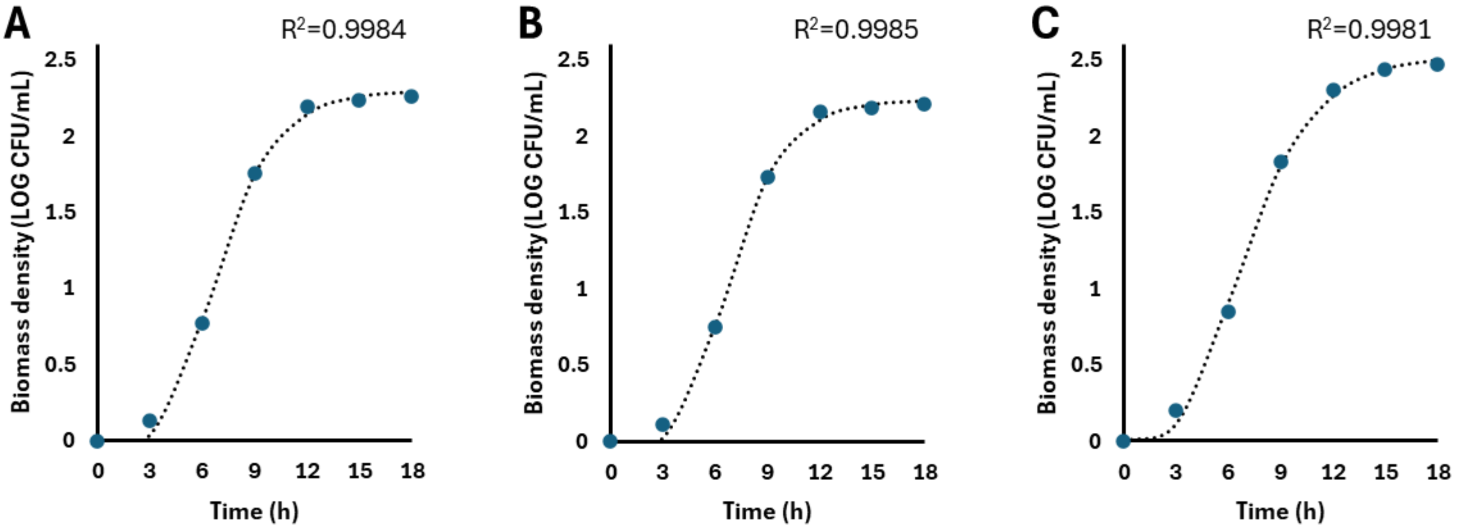


**Figure S2.** Fitting the Gompertz Equation (dotted line) to the growth data of the *K. marxianus* monoculture.

microorganisms

# Extracellular metabolites from *Saccharomyces cerevisiae* modulate the growth and fermentative performance of *Kluyveromyces marxianus*

**Jairo Gallardo-Rivera, Oscar E. Soto-Malpica, Erick D. Acosta-García, Perla G. Vázquez-Ortega, Juan A. Rojas-Contreras and Nicolas O. Soto-Cruz ***

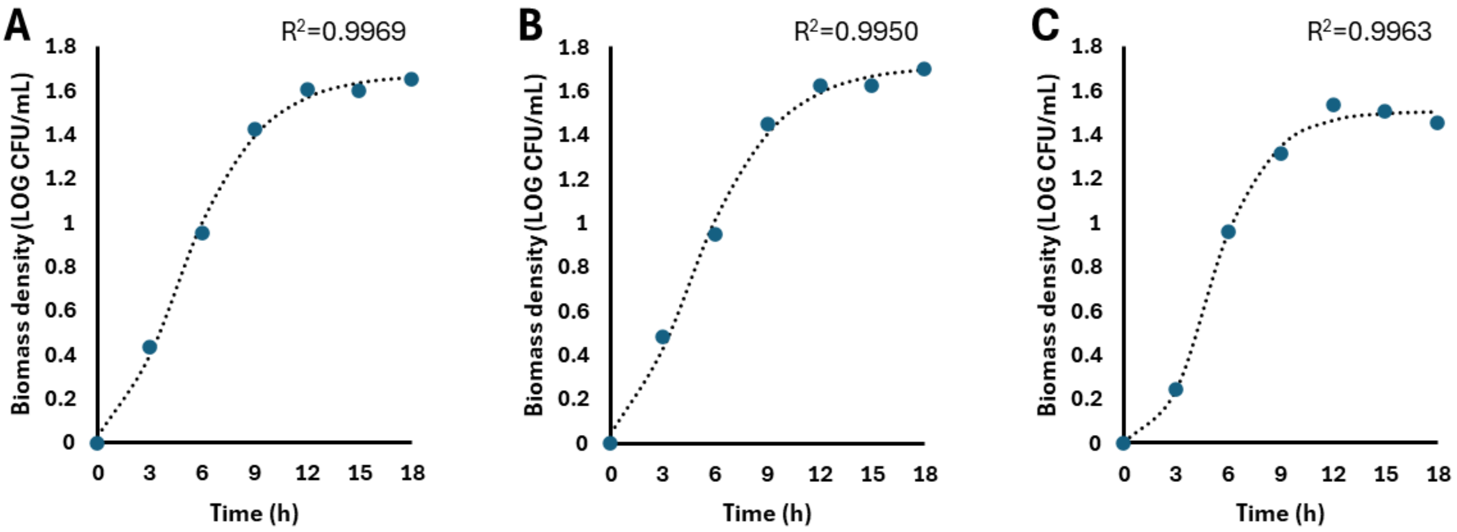


**Figure S3.** Fitting the Gompertz Equation (dotted line) to the growth data of the *S. cerevisiae* in coculture.

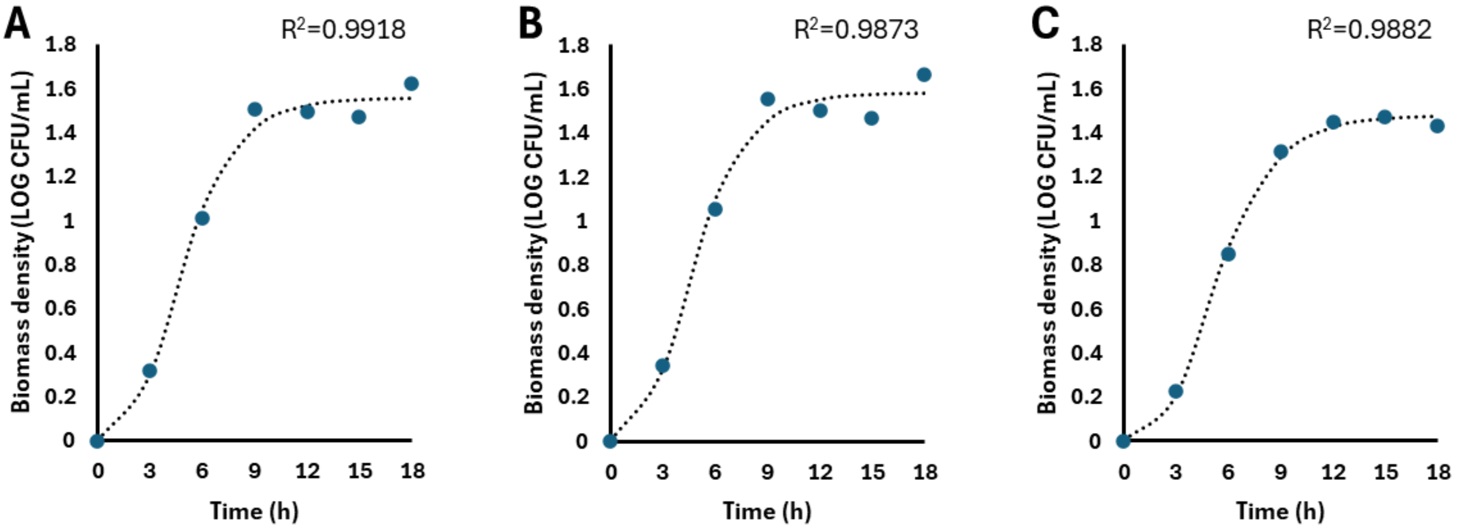


**Figure S4.** Fitting the Gompertz Equation (dotted line) to the growth data of the *K. marxianus* in coculture.

microorganisms

MDPI

# Extracellular metabolites from *Saccharomyces cerevisiae* modulate the growth and fermentative performance of *Kluyveromyces marxianus*

**Jairo Gallardo-Rivera, Oscar E. Soto-Malpica, Erick D. Acosta-García, Perla G. Vázquez-Ortega, Juan A. Rojas-Contreras and Nicolas O. Soto-Cruz ***

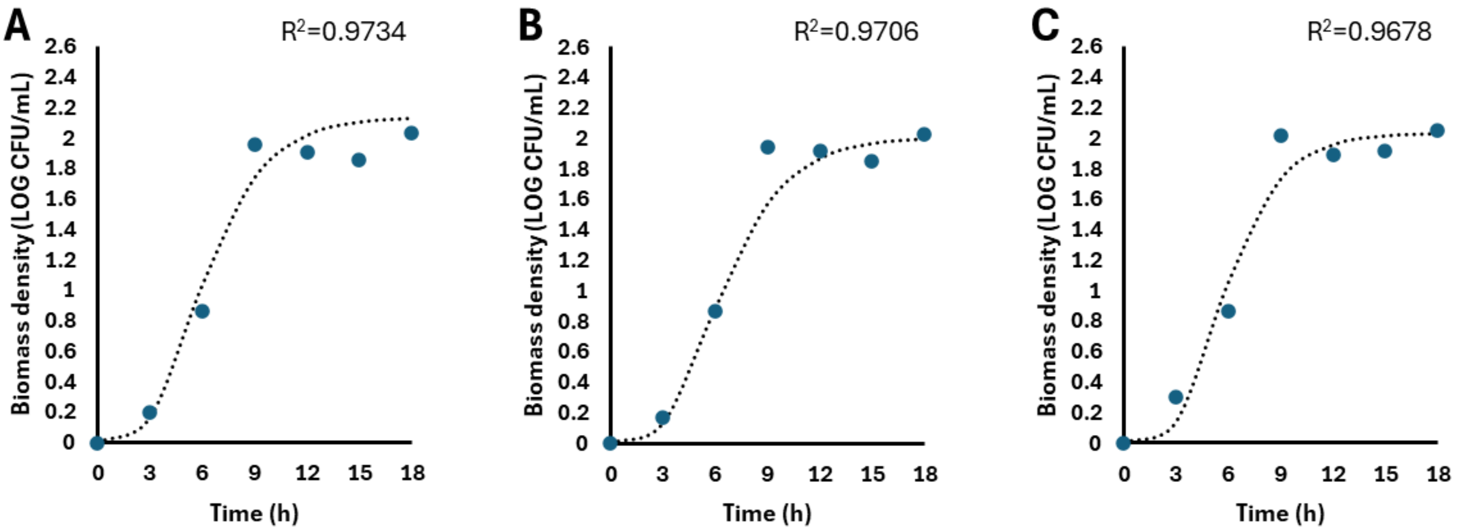


**Figure S5.** Fitting the Gompertz Equation (dotted line) to the growth data of the *K.marxianus* on the cell-free medium previously fermented by *Saccharomyces cerevisiae* for 6 h.

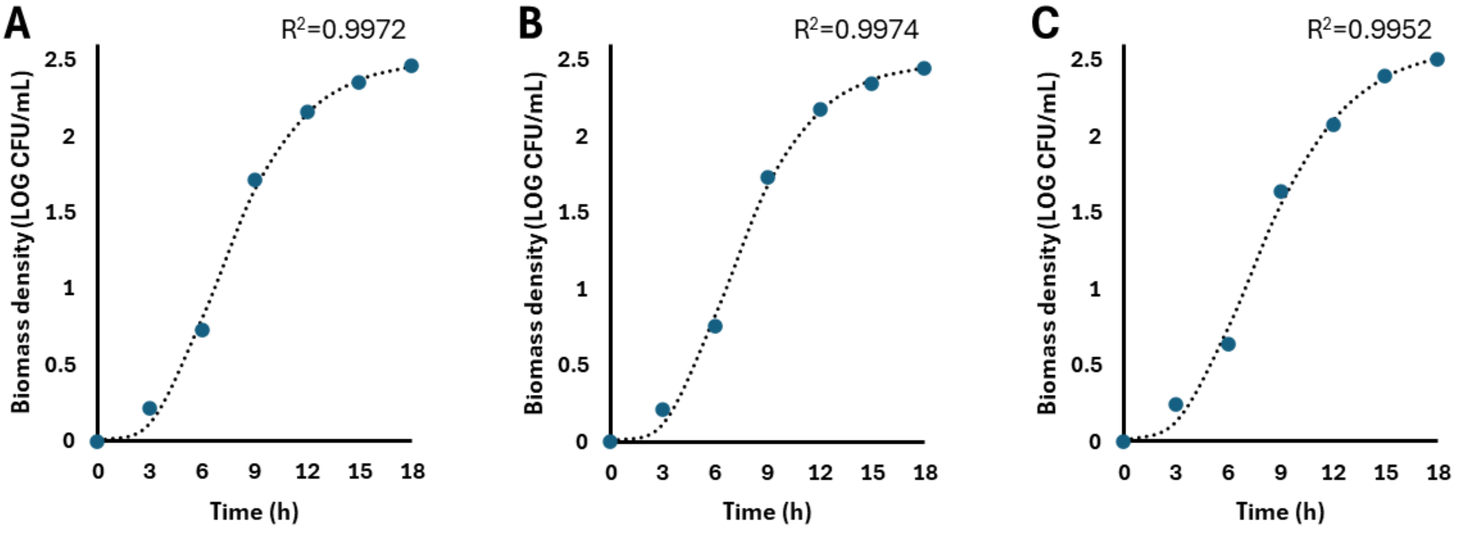


**Figure S6.** Fitting the Gompertz Equation (dotted line) to the growth data of the *K.marxianus* on the cell-free medium previously fermented by *Saccharomyces cerevisiae* for 17.5 h.

microorganisms

MDPI

# Extracellular metabolites from *Saccharomyces cerevisiae* modulate the growth and fermentative performance of *Kluyveromyces marxianus*

**Jairo Gallardo-Rivera, Oscar E. Soto-Malpica, Erick D. Acosta-García, Perla G. Vázquez-Ortega, Juan A. Rojas-Contreras and Nicolas O. Soto-Cruz ***

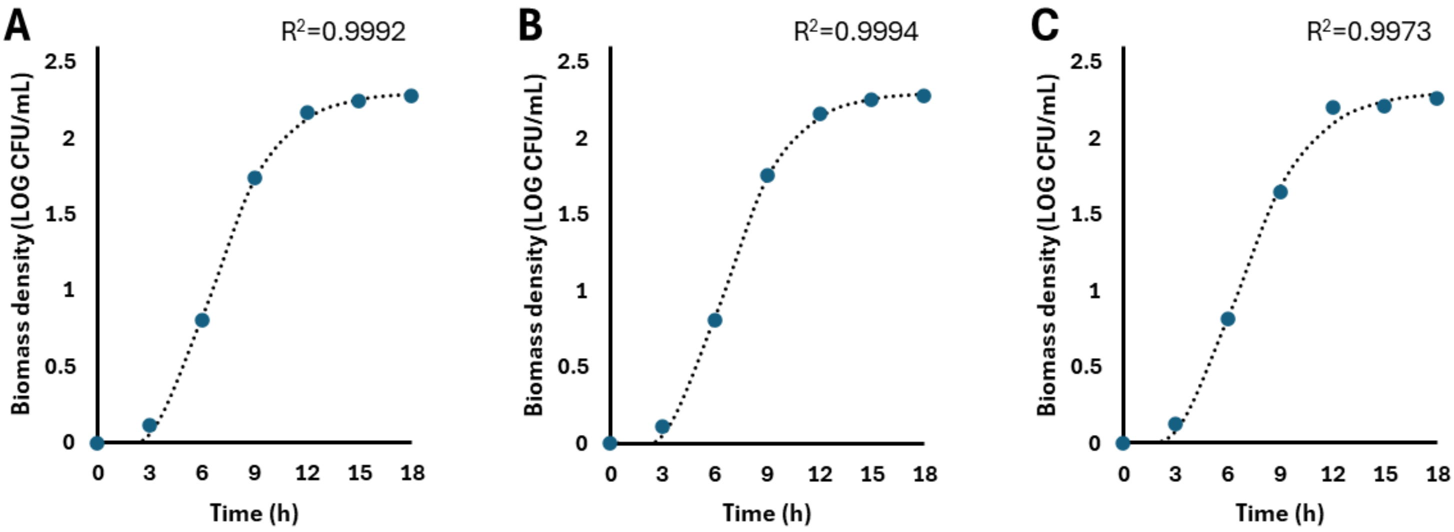


**Figure S7.** Fitting the Gompertz Equation (dotted line) to the growth data of the *K.marxianus* on the cell-free medium previously fermented by *Saccharomyces cerevisiae* for 24 h.

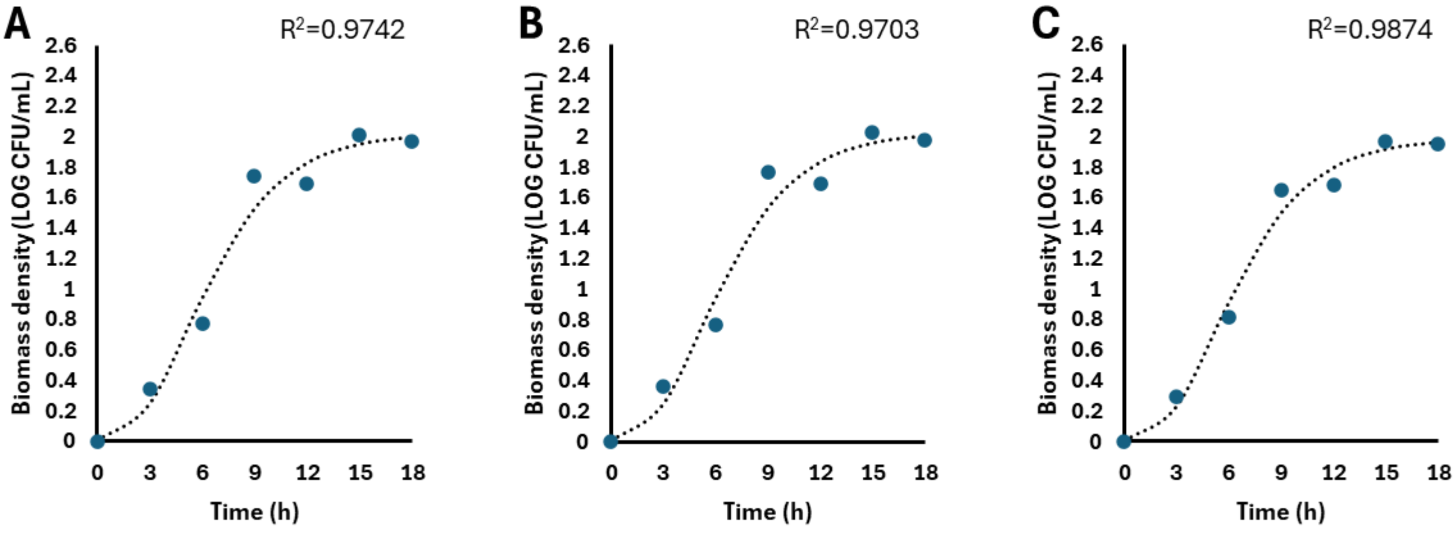


**Figure S8.** Fitting the Gompertz Equation (dotted line) to the growth data of the *K.marxianus* in a cross-exposure assay from 6 to 9 h.

microorganisms

# Extracellular metabolites from *Saccharomyces cerevisiae* modulate the growth and fermentative performance of *Kluyveromyces marxianus*

**Jairo Gallardo-Rivera, Oscar E. Soto-Malpica, Erick D. Acosta-García, Perla G. Vázquez-Ortega, Juan A. Rojas-Contreras and Nicolas O. Soto-Cruz ***

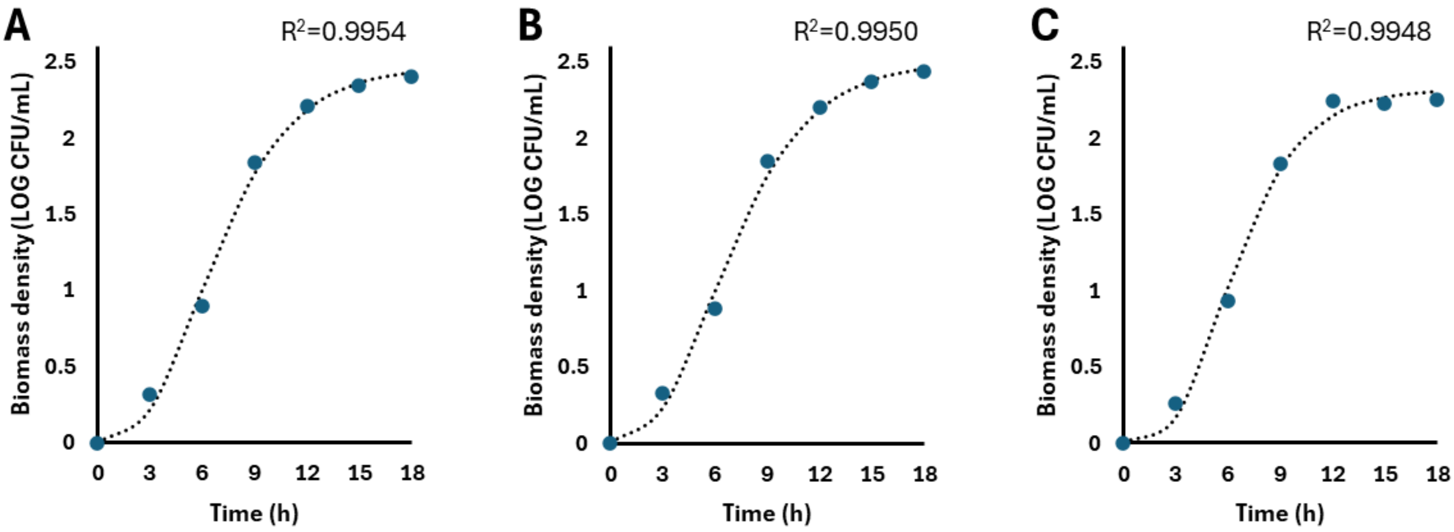


**Figure S9.** Fitting the Gompertz Equation (dotted line) to the growth data of the *K.marxianus* in a cross-exposure assay from 17.5 to 20.5 h.

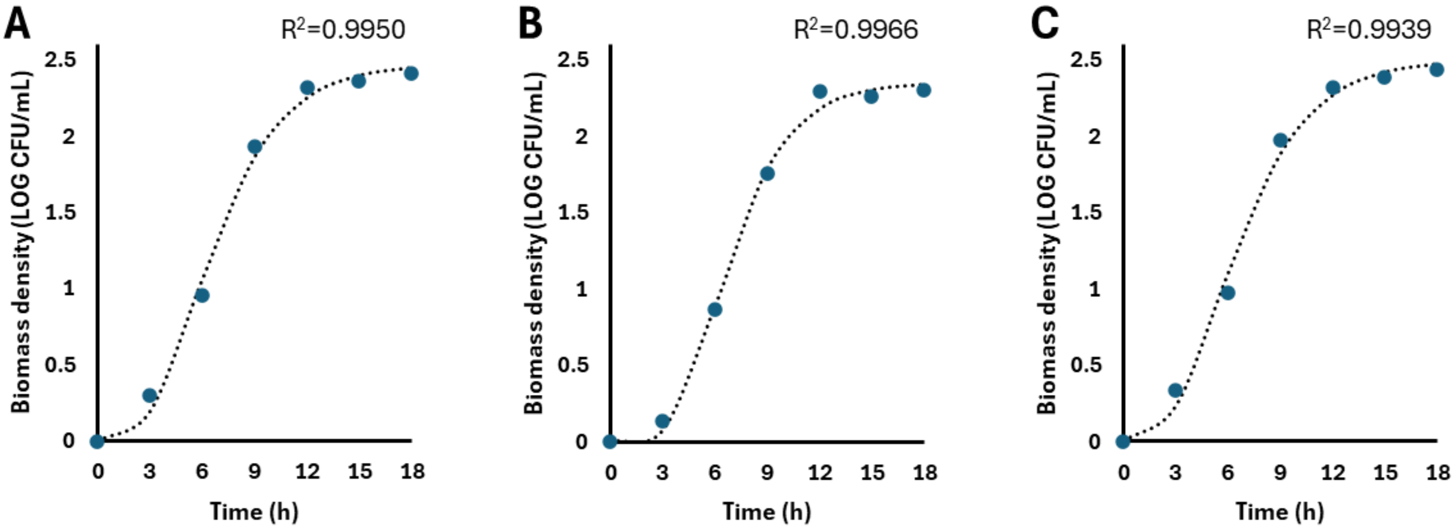


**Figure S10.** Fitting the Gompertz Equation (dotted line) to the growth data of the *K.marxianus* in a cross-exposure assay from 24 to 27 h.
