# Supplementary material for: Extracellular Metabolites from Saccharomyces cerevisiae Modulate the Growth and Fermentative Performance of Kluyveromyces marxianus
Source: Microorganisms. 2026 Apr 16;14(4):890. doi: 10.3390/microorganisms14040890 (PMC13118812; doi:10.3390/microorganisms14040890)
Supplement: Supplementary file 1 [file microorganisms-14-00890-s001.zip › Supplementary_Table_S1.pdf]

microorganisms

MDPI

Extracellular metabolites from *Saccharomyces cerevisiae* modulate the growth and fermentative performance of *Kluyveromyces marxianus*

Jairo Gallardo-Rivera, Oscar E. Soto-Malpica, Erick D. Acosta-García, Perla G. Vázquez-Ortega, Juan A. Rojas-Contreras and Nicolas O. Soto-Cruz *

Table S1. Semi-quantification of volatile compounds (mg/L). Data are presented as mean ± standard deviation of triplicate determinations. Different letters within the same row indicate significant differences among fermentation times (one-way ANOVA followed by Tukey's test, $p < 0.05$). RI_AMDIS: retention index of each compound determined using AMDIS software under the same analytical conditions. RI_L: retention index of each compound previously reported in the literature (* indicates retention index obtained using a DB-Wax column). ND: not detected. UM2: unfermented M2 medium; 6 h: M2 medium fermented by *S. cerevisiae* for 6 h; 17.5 h: M2 medium fermented by *S. cerevisiae* for 17.5 h; 24 h: M2 medium fermented by *S. cerevisiae* for 24 h.

Compounds	RI AMDIS	RIL	Semi-quantification (mg/L)			
			UM2	6 h	17.5 h	24 h
**Acetates**						
**Ethyl acetate**	881.3	888	ND	ND	0.5756 ± 0.0805 [b]	1.1722 ± 0.3915 [a]
**Isoamyl acetate**	1123.1	1122	0.0018 ± 0.0027 [b]	0.0046 ± 0.0048 [b]	0.8984 ± 0.0847 [a]	0.7826 ± 0.0763 [a]
**2-Phenylethyl acetate**	1827.8	1820	0.4861 ± 0.0467 [b]	0.3370 ± 0.1765 [b]	7.5792 ± 1.2282 [a]	7.6221 ± 1.5714 [a]
**Ethyl hexanoate**	1240.8	1235	ND	ND	1.3347 ± 0.0880 [b]	1.5800 ± 0.1614 [a]
**Ethyl octanoate**	1437.6	1431	0.0055 ± 0.0079 [b]	0.0048 ± 0.0058 [b]	0.7269 ± 0.1570 [a]	0.7433 ± 0.1131 [a]
**Ethyl decanoate**	1641.1	1648*	0.0043 ± 0.0039 [c]	0.0226 ± 0.0083 [c]	0.5781 ± 0.1403 [a]	0.2594 ± 0.0696 [b]
**Alcohols**						
**Isobutanol**	1090.2	1096	ND	0.0170 ± 0.0049 [b]	0.3847 ± 0.0475 [a]	0.4013 ± 0.0816 [a]
**Isoamyl alcohol**	1210.4	1210	0.0453 ± 0.0259 [b]	2.9785 ± 2.5796 [b]	24.9851 ± 1.3463 [a]	24.3695 ± 3.8692 [a]
**2-Phenylethanol**	1923	1926	ND	1.7746 ± 0.4126 [b]	53.0510 ± 5.0369 [a]	48.8296 ± 6.2011 [a]
**Methionol**	1723.8	1716	0.0336 ± 0.0171 [b]	0.1504 ± 0.0451 [b]	0.6948 ± 0.1392 [a]	0.4981 ± 0.1084 [a]
**1-Heptanol**	1456.3	1458*	0.0024 ± 0.0021 [c]	0.0084 ± 0.0030 [c]	0.2404 ± 0.0191 [b]	0.2995 ± 0.0335 [a]
**2-Ethyl-1-hexanol**	1489.3	1504	ND	0.0175 ± 0.0039 [b]	0.0726 ± 0.0800 [ab]	0.1676 ± 0.0470 [a]
**1-Dodecanol**	1967.3	1964*	0.0461 ± 0.0059 [b]	0.0536 ± 0.0382 [b]	0.4325 ± 0.2112 [a]	0.5999 ± 0.0699 [a]
**Linalool**	1548.8	1543	ND	0.0088 ± 0.0065 [c]	0.2020 ± 0.0313 [b]	0.2653 ± 0.0139 [a]
**Nerolidol**	2041.4	2040*	ND	0.0054 ± 0.0081 [b]	0.7224 ± 0.1881 [a]	0.8141 ± 0.1985 [a]
**Citronellol**	1767.3	1762	ND	ND	ND	0.1300 ± 0.1142

microorganisms

Extracellular metabolites from *Saccharomyces cerevisiae* modulate the growth and fermentative performance of *Kluyveromyces marxianus*

Jairo Gallardo-Rivera, Oscar E. Soto-Malpica, Erick D. Acosta-García, Perla G. Vázquez-Ortega, Juan A. Rojas-Contreras and Nicolas O. Soto-Cruz *

**Organic acids**						
**Acetic acid**	1466.1	1468	ND	0.0127 ± 0.0086 [a]	0.5307 ± 0.4220 [a]	0.2885 ± 0.1831 [a]
**Hexanoic acid**	1864.6	1867	ND	0.0365 ± 0.0188 [b]	1.1070 ± 0.1144 [a]	0.4450 ± 0.3863 [b]
**Octanoic acid**	2065.3	2067	ND	0.7411 ± 0.3564 [b]	16.7968 ± 2.4606 [a]	13.9375 ± 2.8828 [a]
**Nonanoic acid**	2184.4	2181*	ND	0.2806 ± 0.2848 [a]	0.4882 ± 0.0640 [a]	0.1947 ± 0.0170 [a]
**Decanoic acid**	2279.1	2276	ND	0.4827 ± 0.2439 [b]	13.3535 ± 2.4586 [a]	3.7649 ± 0.4817 [b]
**Aldehydes**						
**2-Methylbutanal**	909	908	ND	0.0024 ± 0.0021	ND	ND
**3-Methylbutanal**	912.8	913	0.6676 ± 0.3581	ND	ND	ND
**Nonanal**	1396.3	1397	0.0328 ± 0.0034 [a]	0.0381 ± 0.0108 [a]	0.1635 ± 0.1001[a]	0.1796 ± 0.1567 [a]
**Decanal**	1501.4	1497	0.0288 ± 0.0126 [b]	0.0503 ± 0.0129 [b]	0.2600 ± 0.0362 [a]	0.2464 ± 0.0182 [a]
**Benzaldehyde**	1533.9	1556	0.3463 ± 0.0292 [a]	0.0434 ± 0.0217 [b]	0.2774 ± 0.0640 [a]	0.2886 ± 0.0822 [a]
**Benzeneacetaldehyde**	1655.8	1651	1.3950 ± 0.1156 [a]	0.0576 ± 0.0636 [b]	0.2711 ± 0.1331 [b]	0.2002 ± 0.1154 [b]
**Methional**	1463.9	1458	0.1298 ± 0.0101	ND	ND	ND
**Furans and furan derivates**						
**Furfural**	1476.1	1493	2.5441 ± 0.2579 [a]	0.1877 ± 0.0687 [b]	0.0469 ± 0.0406 [b]	ND
**2-Acetylfuran**	1515.5	1514*	0.7742 ± 0.0806 [b]	0.8613 ± 0.2271 [b]	2.3445 ± 0.1460 [a]	2.0530 ± 0.3063 [a]
**2-Propionylfuran**	1586.8		0.0447 ± 0.0052 [a]	0.0163 ± 0.0063 [b]	0.0551 ± 0.0036 [a]	0.0495 ± 0.0083 [a]
**2,5-Furandicarboxaldehyde**	1623.1		0.0498 ± 0.0043 [b]	0.0626 ± 0.0194 [b]	0.1268 ± 0.0073 [a]	0.0661 ± 0.0140 [b]
**Furfuryl alcohol**	1669.1	1669*	ND	0.2910 ± 0.0764 [b]	0.4856 ± 0.0667 [a]	0.4339 ± 0.0597 [ab]
**Pyrazines**						
**2,5-Dimethylpyrazine**	1326.2	1325*	0.2093 ± 0.0204 [a]	0.2304 ± 0.0516 [a]	0.3213 ± 0.0332 [a]	0.1501 ± 0.1300 [a]
**2,6-Dimethylpyrazine**	1332.1	1331*	0.0745 ± 0.0049 [a]	0.0674 ± 0.0590 [a]	ND	ND
**Trimethylpyrazine**	1404.5	1403	0.0088 ± 0.0076 [a]	0.0093 ± 0.0083 [a]	ND	ND
**Ketones**						
**(E)-6,10-Dimethyl-5,9-undecadien-2-one**	1861.6	1858*	0.0110 ± 0.0100 [b]	0.0377 ± 0.0364 [b]	0.6047 ± 0.1483 [a]	0.6591 ± 0.1302 [a]
